# Differential Temporal Dynamics of Axial and Appendicular Ataxia in SCA3

**DOI:** 10.1002/mds.29135

**Published:** 2022-07-08

**Authors:** Roderick P.P.W.M. Maas, Steven Teerenstra, Manuela Lima, Paula Pires, Luís Pereira de Almeida, Judith van Gaalen, Dagmar Timmann, Jon Infante, Chiadi Onyike, Khalaf Bushara, Heike Jacobi, Kathrin Reetz, Magda M. Santana, Joana Afonso Ribeiro, Jeannette Hübener‐Schmid, Jeroen J. de Vries, Matthis Synofzik, Ludger Schöls, Hector Garcia‐Moreno, Paola Giunti, Jennifer Faber, Thomas Klockgether, Bart P.C. van de Warrenburg

**Affiliations:** ^1^ Department of Neurology, Donders Institute for Brain, Cognition, and Behaviour Radboud University Medical Center Nijmegen The Netherlands; ^2^ Department for Health Evidence, Biostatistics Section Radboud University Medical Center Nijmegen The Netherlands; ^3^ Faculdade de Ciências e Tecnologia Universidade dos Açores Azores Portugal; ^4^ Instituto de Biologia Molecular e Celular (IBMC), Instituto de Investigação e Inovação em Saúde (i3S) Universidade do Porto Porto Portugal; ^5^ Department of Neurology Hospital Santo Espírito da ilha Terceira Azores Portugal; ^6^ Center for Neuroscience and Cell Biology (CNC) University of Coimbra Coimbra Portugal; ^7^ Center for Innovation in Biomedicine and Biotechnology (CIBB) University of Coimbra Coimbra Portugal; ^8^ Faculty of Pharmacy University of Coimbra Coimbra Portugal; ^9^ Department of Neurology and Center for Translational Neuro‐ and Behavioral Sciences (C‐TNBS), Essen University Hospital University of Duisburg‐Essen Essen Germany; ^10^ Neurology Service, Centro de Investigación Biomédica en Red de Enfermedades Neurodegenerativas (CINERNED), University Hospital Marques de Valdecilla‐IDIVAL University of Cantabria‐UC Santander Spain; ^11^ Department of Psychiatry and Behavioral Sciences Johns Hopkins University School of Medicine Baltimore Maryland USA; ^12^ Ataxia Center, Department of Neurology University of Minnesota Minneapolis Minnesota USA; ^13^ Department of Neurology University Hospital Heidelberg Heidelberg Germany; ^14^ Department of Neurology RWTH Aachen University Aachen Germany; ^15^ JARA‐BRAIN Institute Molecular Neuroscience and Neuroimaging Forschungszentrum Jülich GmbH and RWTH Aachen University Aachen Germany; ^16^ Department of Neurology, Child Development Centre Coimbra's Hospital and University Centre Coimbra Portugal; ^17^ Institute of Medical Genetics and Applied Genomics University of Tübingen Tübingen Germany; ^18^ Department of Neurology, University Medical Center Groningen University of Groningen Groningen The Netherlands; ^19^ Expertise Center Movement Disorders Groningen University Medical Center Groningen Groningen The Netherlands; ^20^ Department of Neurodegenerative Diseases, Center for Neurology and Hertie Institute for Clinical Brain Research University of Tübingen Tübingen Germany; ^21^ German Center for Neurodegenerative Diseases (DZNE) Tübingen Germany; ^22^ Ataxia Centre, Department of Clinical and Movement Neurosciences, UCL Queen Square Institute of Neurology University College London London United Kingdom; ^23^ Department of Neurogenetics, National Hospital for Neurology and Neurosurgery University College London Hospital NHS Foundation Trust London United Kingdom; ^24^ Department of Neurology University Hospital Bonn Bonn Germany; ^25^ German Center for Neurodegenerative Diseases (DZNE) Bonn Germany

**Keywords:** spinocerebellar ataxia type 3, natural history, Scale for the Assessment and Rating of Ataxia, disease progression

## Abstract

**Background:**

Disease severity in spinocerebellar ataxia type 3 (SCA3) is commonly defined by the Scale for the Assessment and Rating of Ataxia (SARA) sum score, but little is known about the contributions and progression patterns of individual items.

**Objectives:**

To investigate the temporal dynamics of SARA item scores in SCA3 patients and evaluate if clinical and demographic factors are differentially associated with evolution of axial and appendicular ataxia.

**Methods:**

In a prospective, multinational cohort study involving 11 European and 2 US sites, SARA scores were determined longitudinally in 223 SCA3 patients with a follow‐up assessment after 1 year.

**Results:**

An increase in SARA score from 10 to 20 points was mainly driven by axial and speech items, with a markedly smaller contribution of appendicular items. Finger chase and nose‐finger test scores not only showed the lowest variability at baseline, but also the least deterioration at follow‐up. Compared with the full set of SARA items, omission of both tests would result in lower sample size requirements for therapeutic trials. Sex was associated with change in SARA sum score and appendicular, but not axial, subscore, with a significantly faster progression in men. Despite considerable interindividual variability, the average annual progression rate of SARA score was approximately three times higher in subjects with a disease duration over 10 years than in those within 10 years from onset.

**Conclusion:**

Our findings provide evidence for a difference in temporal dynamics between axial and appendicular ataxia in SCA3 patients, which will help inform the design of clinical trials and development of new (etiology‐specific) outcome measures. © 2022 The Authors. *Movement Disorders* published by Wiley Periodicals LLC on behalf of International Parkinson and Movement Disorder Society.

Spinocerebellar ataxia type 3 (SCA3) is a devastating neurodegenerative disorder that principally affects the deep cerebellar and pontine nuclei, basal ganglia, and spinal cord.^1^ Despite substantial geographic variation in prevalence rates, it is considered the most common form of dominantly inherited ataxia worldwide, accounting for an estimated 20% to 50% of affected families.^2^, ^3^, ^4^ SCA3 is caused by the expansion of an unstable polyglutamine‐encoding CAG repeat in the *ATXN3* gene, which triggers an intricate series of events that culminate in widespread neuronal loss.^5^ In parallel with these pathological changes, mutation carriers have been shown to exhibit a significantly shorter survival time than their asymptomatic relatives, with death occurring after a mean disease duration of ~21 years.^6^

Quantification of ataxia severity and natural disease progression in SCAs constitutes an essential prerequisite for objectively determining the effectiveness of therapies in future randomized controlled trials. The last 15 years have witnessed important developments in the field, including the construction and validation of the Scale for the Assessment and Rating of Ataxia (SARA), definition of a preclinical disease stage, establishment of European and American research consortia, and implementation of large‐scale longitudinal studies.^7^, ^8^, ^9^, ^10^, ^11^ These efforts, however, mainly focused on progression of overall ataxia severity, as captured by annual change in SARA sum score, and did not specifically assess the temporal dynamics of single SARA items or subscores grouping axial versus appendicular items.

Differences in the natural history of axial and appendicular signs have recently been described in Friedreich ataxia and were also noticed in a single‐center study involving SCA3 patients.^12^, ^13^ A careful investigation of item scores could not only provide more detailed information about the clinical evolution of degenerative cerebellar diseases, but might also have important implications for the application of SARA as (primary) outcome measure in therapeutic trials. Using a combination of cross‐sectional and longitudinal data from a large international cohort of SCA3 mutation carriers, we sought to investigate the progression pattern of SARA and its individual items, with specific attention to axial versus appendicular subscores. Furthermore, we examined whether demographic, clinical, and genetic factors differentially covary with progression rates of axial and appendicular subscores and whether annual changes in SARA item scores correlate with changes in corresponding functional measures in SCA3.

## Methods

### Study Design and Participants

The European Spinocerebellar ataxia type 3/Machado‐Joseph disease Initiative (ESMI) is a prospective observational multicenter study that aims to comprehensively delineate disease progression with standardized clinical assessments (see below), magnetic resonance imaging (MRI) scans, and peripheral blood and cerebrospinal fluid biomarkers. Baseline data were collected between November 2016 and March 2020 at the participating centers in Coimbra, the Azores, London, Bonn, Tübingen, Groningen, Nijmegen, Essen, Santander, Heidelberg, and Aachen and, additionally, at 2 United States (US) sites in Minneapolis and Baltimore. In the present investigation, we focused on clinical measures in manifest disease and examined cross‐sectional and longitudinal data from ataxic individuals with SARA scores between 3 and 30. The latter cut‐off value was chosen because only 8 out of 231 patients in the cohort had sum scores between 30 and 40, precluding robust inferences for this late disease stage. Longitudinal results are derived from patients with a complete set of SARA ratings at baseline and 1‐year follow‐up (± 3 months), which matches the duration of several previous or ongoing trials and the expected duration of (initial) future therapeutic trials.^14^, ^15^, ^16^

The study was approved by the ethics committees of contributing centers and written informed consent was obtained from each participant at enrolment.

### Procedures

SARA is used as the primary clinical outcome measure in ESMI to track progression of ataxia severity. The scale contains 8 items, which together yield a sum score between 0 (absence of ataxia) and 40 (most severe ataxia).^11^ SARA ratings at each visit were determined by trained and experienced investigators. In 8 out of 13 centers, patients were seen by the same investigator at baseline and follow‐up. Single items were combined in relevant functional domains by aggregating gait, stance, and sitting into SARA axial (maximum 18 points), finger chase, nose‐finger test, and fast alternating hand movements into SARA upper limb (maximum 12 points), and the three upper limb items and heel‐shin slide into SARA appendicular (maximum 16 points).^17^, ^18^ In addition to SARA, we used the 8 m walk test (8MWT), nine‐hole peg test (9HPT), and PATA repetition task, which collectively comprise the SCA Functional Index (SCAFI), as measures of gait speed, manual dexterity, and articulation speed, respectively.^19^ Two consecutive trials of each test were conducted and mean scores were calculated. In keeping with SCAFI instructions, trials were excluded when time required to walk 8 m exceeded 180 seconds and time required to complete the 9HPT exceeded 300 seconds.^19^ Extracerebellar involvement was quantified through the Inventory of Non‐Ataxia Signs (INAS) count, which ranges from 0 to 16.^20^ Finally, disease duration was computed by subtracting the age at which first gait difficulties appeared (by a patient's own report) from the age at baseline assessment.

### Statistical Analysis

#### Cross‐Sectional Analyses on Baseline Data

Relationships between disease duration, SARA sum score, and relative contributions of axial and appendicular subscores were evaluated using Spearman's rank‐order correlation coefficients. Because the number of response options differs across the 8 items, we examined to what extent the overall score and item scores at baseline aligned. To this end, one‐sample *t* tests were applied to compare observed contributions of every item to SARA sum score with theoretically expected contributions (here, “expected” refers to the quotient of maximum item score and maximum sum score; eg, gait 8/40 = 0.20). Single SARA items and aggregated subscores were further investigated in relation to SARA sum score with local polynomial (LOESS) regression, which is a more flexible technique than simple ordinary least squares regression. To quantify the (non‐linear) dynamics of each item and enable statistical analyses, patients were grouped in 5 bins of equal width according to SARA sum score (ie, 3–8, 8.5–13.5, 14–19, 19.5–24.5, and 25–30). Differences in item scores between patients in consecutive bins were ascertained using analysis of variance with Tukey or Games‐Howell post hoc tests, depending on whether or not the assumption of homogeneity of variance had been met.

#### Longitudinal Analyses

χ^2^ tests and *t* tests were applied to investigate whether sex, age, disease duration, SARA score, and aggregated subscores differed between patients who only had a baseline visit from those who returned for follow‐up.

Standardized response means (SRMs) were calculated for SARA, 8MWT, 9HPT, and the PATA repetition task. In line with the EUROSCA study, values of 0.20, 0.50, and 0.80 were considered to indicate small, moderate, and large changes in terms of statistical variation.^21^

Associations between changes in SARA item scores and changes in corresponding SCAFI tests were determined using Spearman's rank‐order correlation coefficients.

Multivariable linear regression analyses with backward selection were performed to identify clinical, demographic, and genetic factors that might affect progression of axial and appendicular subscores and SARA sum score. The respective baseline SARA (sub)score, disease duration, age, sex, repeat length of the expanded allele, utilization of physical therapy, and INAS count were selected as independent variables. Unpaired *t* tests were subsequently used to compare progression of SARA items and aggregated subscores between male and female patients.

Finally, based on the annual progression rate of SARA score, sample sizes for future clinical trials in SCA3 patients were calculated, assuming a power of 0.8 or 0.9, α of 0.05, and a range of possible interventional effects (ie, expected reductions in natural progression rate from 0.1 to 1.0 with steps of 0.1). Because targeted molecular therapies are anticipated to have the largest benefits in terms of halting further progression when administered early in the disease course, a separate sample size calculation was conducted for mildly affected patients with baseline SARA scores between 3 and 10.

Statistical analyses were performed in SPSS Statistics (IBM, version 25).

## Results

### Demographic and Clinical Characteristics of Participants

Baseline data were collected from 223 SCA3 patients (114 males, 51.1%) with a mean age of 51.2 years (standard deviation [SD], 11.2 years), disease duration of 11.6 years (SD, 6.9 years), SARA score of 13.8 points (SD, 7.3 points), and repeat length of 68.6 (SD, 4.0). Clinical outcome measures were available as follows: SARA score 100%, 8MWT 65.9%, 9HPT dominant hand 90.1%, 9HPT non‐dominant hand 89.2%, and PATA repetition task 93.3%. Follow‐up visits after 1 year were completed by 156 patients, with clinical outcome measures being available as follows: SARA score 100%, 8MWT 66.0%, 9HPT dominant hand 90.4%, 9HPT non‐dominant hand 88.5%, and PATA repetition task 94.2%. Of these 156 individuals, 96 (61.5%) were treated by a physical therapist, whereas 53 (34.0%) were not. Data from the remaining 7 patients (4.5%) were missing. There were no significant differences in demographic and clinical characteristics between subjects who only had a baseline visit and those who returned for follow‐up (Supplementary Table S1).

### Cross‐Sectional Data

#### Contributions of Single Items to SARA Score and Influence of Disease Duration

Based on disease duration, the estimated mean increase in SARA score was 1.46 points per year (SD, 0.84). Estimated mean annual increases in axial and appendicular subscores were 0.76 (SD, 0.44) and 0.51 (SD, 0.37) points, respectively.

Baseline SARA score was composed as follows: gait 4.03 (SD, 2.20), stance 2.60 (SD, 1.72), sitting 0.77 (SD, 0.88), speech 1.75 (SD, 1.29), finger chase 1.06 (SD, 0.65), nose‐finger test 0.74 (SD, 0.63), fast alternating hand movements 1.25 (SD, 0.90), and heel‐shin slide 1.65 (SD, 0.91). There were notable discrepancies between theoretically expected and observed contributions for all items (*P* < 0.001 except fast alternating hand movements [*P* = 0.025]) (Supplementary Table S2). Gait and stance were responsible for nearly 50% of SARA score, while sitting and nose‐finger test contributed least, also in relative sense (ie, when taking into account the number of response levels per item). Finger chase and nose‐finger test scores had the smallest SDs, also in relative terms, indicating that these items show the least variability. In fact, 70.0% and 84.3% of patients, respectively, had a score of 1 or lower, while ratings higher than 2 were rare (only 2.2% of participants at finger chase and 1.3% at the nose‐finger test).

**FIG. 1:**
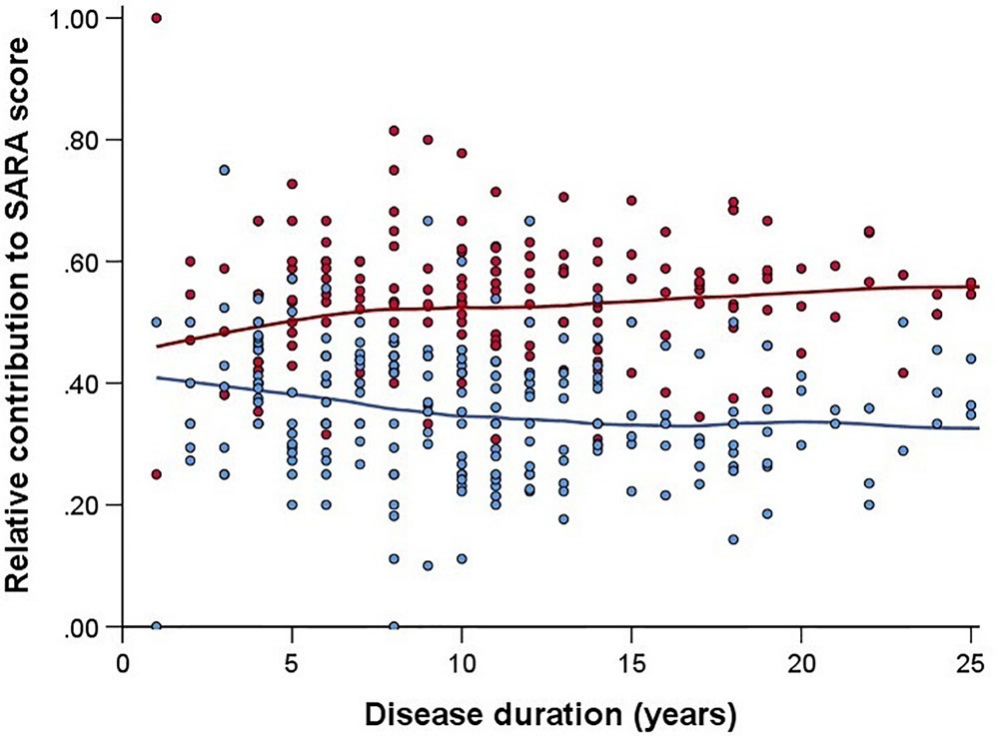
Relative contributions of axial and appendicular SARA subscores at baseline versus disease duration in SCA3 patients, fitted with LOESS regression. Red dots and the red line represent axial subscores, whereas blue dots and the blue line represent appendicular subscores. [Color figure can be viewed at wileyonlinelibrary.com]

Axial and appendicular subscores were 7.40 (SD, 4.43) and 4.69 (SD, 2.34), respectively. Relative contributions of axial items tended to increase in parallel with disease duration (ρ = 0.15, *P* = 0.03), whereas those of appendicular items decreased (ρ = −0.19, *P* = 0.005) (Fig. 1).

#### SARA Items Versus SARA Sum Score

**FIG. 2:**
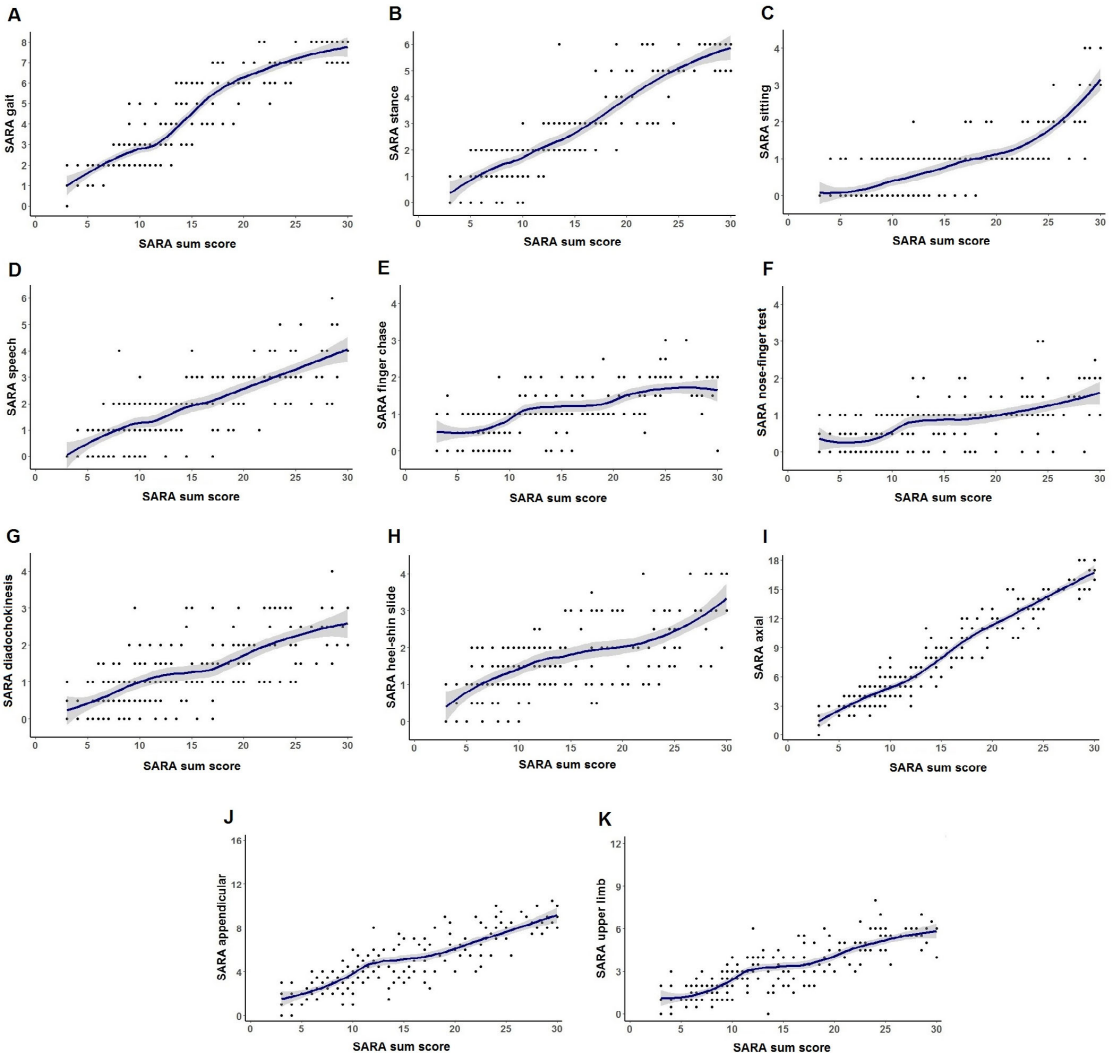
Single SARA items (**A**–**H**) and aggregated subscores (**I**–**K**) in relation to SARA sum score in SCA3 patients, fitted with LOESS regression. [Color figure can be viewed at wileyonlinelibrary.com]

Relationships between single SARA items or aggregated subscores and SARA sum score are illustrated in Figure 2. For the items of gait, stance, sitting, and speech, there were significant differences (*P* < 0.0125) between patients in consecutive 5‐point SARA sum score bins (Supplementary Table S3). A linear relationship with SARA score was observed for the gait, stance, and speech items, whereas sitting scores showed less variability (most often 0 or 1 over a broad range of SARA sum scores) and an exponential progression pattern. In contrast to these first 4 items, ratings at finger chase, nose‐finger test, fast alternating hand movements, and heel‐shin slide did not significantly differ between patients who had a SARA sum score of 8.5–13.5 versus 14–19 (*P* > 0.0125), which is visualized as a plateau in the respective graphs (Fig. 2E–H). Similarly, post hoc comparisons showed no significant differences in scores at these 4 items between individuals with a SARA score of 19.5–24.5 and 25–30 (Supplementary Table S3).

### Longitudinal Data

#### Changes in SARA Score and Contributions of Single Items

Compared with their baseline visit, 113 patients (72.4%) had a higher SARA score at 1‐year follow‐up, 13 (8.3%) had an identical score, and 30 (19.2%) had a lower score, yielding a mean annual increase of 1.50 points (SD, 2.85). Lower scores after 1 year were particularly observed at nose‐finger (31.4%) and fast alternating hand movements items (23.1%). Mean annual change in SARA sum score was composed as follows: gait 0.24 (SD, 0.87), stance 0.27 (SD, 1.04), sitting 0.27 (SD, 0.73), speech 0.20 (SD, 0.72), finger chase 0.11 (SD, 0.60), nose‐finger test −0.02 (SD, 0.65), fast alternating hand movements 0.21 (SD, 0.76), and heel‐shin slide 0.23 (SD, 0.76). Relative contributions of items were thus more or less similar, notable exceptions being the nose‐finger and finger chase tests (Table 1). As shown in the right columns of this table, contributions of both tests decreased further after exclusion of 15 patients who had already attained a maximum score at one or more items at baseline (mostly at gait and/or stance). Correlations between changes in SARA item scores and changes in corresponding SCAFI tests are described in the Supporting Data.

**TABLE 1:** Annual changes and relative contributions of single and aggregated SARA item scores to delta SARA sum score in SCA3 patients

	Annual change (n = 156)		Annual change (n = 141)	
	Mean ± SD	% of total	Mean ± SD	% of total
Single items				
Gait	0.24 ± 0.87	15.8	0.25 ± 0.90	17.1
Stance	0.27 ± 1.04	17.9	0.32 ± 0.98	22.0
Sitting	0.27 ± 0.73	17.9	0.24 ± 0.72	16.6
Speech	0.20 ± 0.72	13.2	0.17 ± 0.72	11.7
Finger chase	0.11 ± 0.60	7.0	0.07 ± 0.55	4.9
Nose‐finger test	−0.02 ± 0.65	−1.1	−0.04 ± 0.62	−2.4
Diadochokinesia	0.21 ± 0.76	14.1	0.20 ± 0.75	13.7
Heel‐shin slide	0.23 ± 0.76	15.1	0.24 ± 0.69	16.6
Aggregated subscores				
Axial	0.78 ± 1.80	51.6	0.81 ± 1.80	55.6
Upper limb	0.30 ± 1.31	20.0	0.23 ± 1.29	16.1
Appendicular	0.53 ± 1.65	35.2	0.48 ± 1.55	32.7
Total	1.50 ± 2.85	100	1.45 ± 2.85	100

A separate analysis (right columns) was conducted after exclusion of 15 patients who had already attained a maximum score at one or more items at baseline (mostly gait and/or stance).

Only SARA reached the SRM criterion of moderate effect size (0.53), while small effects were found for the 8MWT (0.38) and 9HPT (0.24). The SRM of 0.063 for the PATA repetition task was negligible.

#### Influence of Demographic, Clinical, and Genetic Factors on Ataxia Progression

Using multivariable linear regression models, we examined the influence of age, sex, repeat length of the expanded allele, disease duration, number of extracerebellar signs, utilization of physical therapy, and ataxia severity (ie, either axial, appendicular, or SARA sum score) on progression of axial and appendicular subscores and SARA sum score. Of these variables, only sex was independently associated with change in SARA sum score (*b* = 1.06, SE = 0.48, *P* = 0.029). Although there were no differences between male and female patients in age (*P* = 0.30), disease duration (*P* = 0.27), and SARA score (*P* = 0.63) at baseline, the annual increase in men (2.02 ± 2.78) was, on average, more than twice as high as that in women (0.96 ± 2.85). However, despite being the only significant predictor, sex explained just 3.5% of the variance in delta SARA score, suggesting a large influence of other factors not covered in the model. Discordance between both sexes was also observed in the annual change in axial and appendicular subscores (Table 2). The former comprised, by far, the largest proportion of delta SARA score in women, whereas contributions of both subscores were nearly equal in men. Annual increase in appendicular subscore was predicted by sex (*b* = 0.69, SE = 0.27, *P* = 0.011), baseline appendicular subscore (*b* = −0.21, SE = 0.07, *P* = 0.002), and disease duration (*b* = 0.058, SE = 0.02, *P* = 0.011), which together accounted for 12% of its variance. Finally, baseline axial subscore (*b* = −0.11, SE = 0.05, *P* = 0.014), disease duration (*b* = 0.069, SE = 0.03, *P* = 0.014), and repeat length of the expanded allele (*b* = 0.073, SE = 0.04, *P* = 0.08), but not sex, affected the annual increase in axial subscore, explaining 6% of its variance.

**TABLE 2:** Annual change in single SARA item scores and aggregated subscores in male and female SCA3 patients

	Sex				
	Male (n = 80)		Female (n = 76)		Difference
	Mean ± SD	% of total	Mean ± SD	% of total	*P* value
Single items					
Gait	0.30 ± 0.85	14.9	0.17 ± 0.90	17.7	0.36
Stance	0.35 ± 0.87	17.3	0.18 ± 1.20	18.8	0.32
Sitting	0.31 ± 0.76	15.3	0.22 ± 0.70	22.9	0.45
Speech	0.19 ± 0.77	9.4	0.21 ± 0.68	21.9	0.84
Finger chase	0.11 ± 0.65	5.4	0.11 ± 0.55	11.5	0.99
Nose‐finger test	0.04 ± 0.63	2.0	−0.08 ± 0.66	−8.3	0.24
Diadochokinesia	0.34 ± 0.73	16.8	0.08 ± 0.77	8.3	0.033
Heel‐shin slide	0.38 ± 0.71	18.8	0.07 ± 0.78	7.3	0.009
Aggregated subscores					
Axial	0.96 ± 1.62	47.5	0.58 ± 1.97	60.4	0.19
Upper limb	0.49 ± 1.38	24.3	0.11 ± 1.22	11.5	0.069
Appendicular	0.87 ± 1.69	43.1	0.17 ± 1.53	17.7	0.008
Total	2.02 ± 2.78	100	0.96 ± 2.85	100	0.02

Plots of the relationships between disease duration, baseline ataxia severity, and disease progression show a large amount of interindividual variability (Supplementary Fig. **S1**). Nonetheless, the average annual progression rate of SARA score was approximately three times higher in SCA3 patients with a disease duration over 10 years (2.24 ± 2.41) than in those within 10 years from onset (0.78 ± 3.13, *P* = 0.002).

#### Sample Size Calculations for Therapeutic Trials

**FIG. 3:**
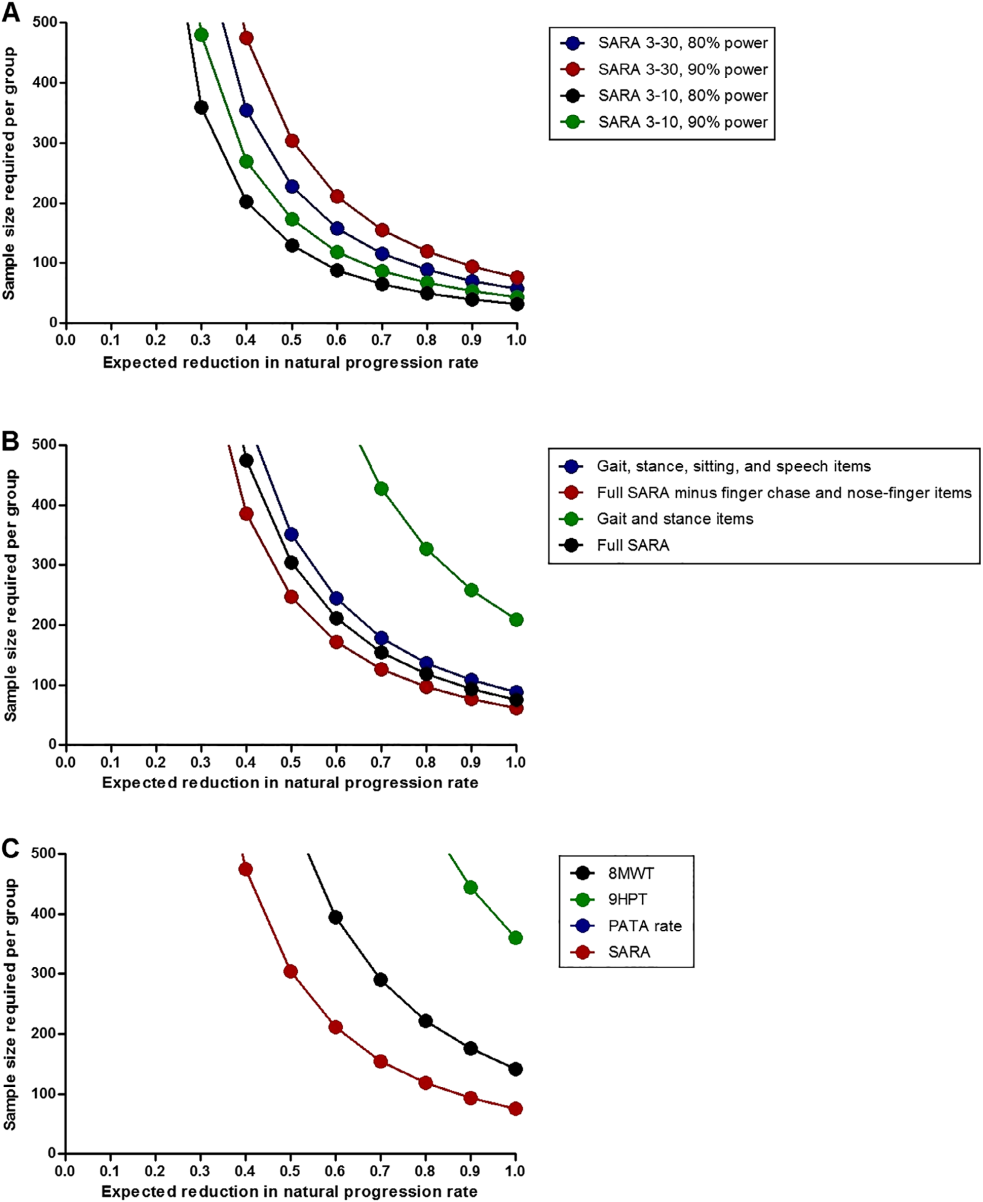
Required numbers of SCA3 patients per group in two‐arm therapeutic trials for a range of possible interventional effects when using SARA sum score (**A**), various subsets of SARA items (**B**), or SCAFI tests (**C**) as the primary endpoint. (**A**) The inclusion of mildly affected patients with SARA scores between 3 and 10 would lower the required number of patients. (**B**) Compared with the full set of SARA items (red circles), omission of finger chase and nose‐finger tests (black circles) would lead to a reduction in sample size, whereas combinations of gait, stance, sitting, and speech items (blue circles) and gait and stance items (green circles) would require a higher number of participants. (**C**) A considerably larger sample size is needed when using the 8MWT, 9HPT, or PATA repetition task as the primary endpoint. Note that the line for PATA repetition rate is missing in the figure as much more than 500 SCA3 patients would be required. A power of 0.9 and α level of 0.05 are assumed in panels (**B**) and (**C**). [Color figure can be viewed at wileyonlinelibrary.com]

Based on the natural history, as outlined above, we determined sample sizes for therapeutic trials in SCA3 patients, assuming a power of 0.9, α of 0.05, follow‐up duration of 1 year, and varying effect sizes (Fig. 3). These analyses showed that 304 individuals are needed per group in order to be able to detect a 50% reduction in progression of SARA score. Should a trial only include patients with a SARA score between 3 and 10 (mean annual increase ± SD, 1.51 ± 2.15 points), the required number per group decreases to 173. We subsequently evaluated whether selection of a subset of items would lead to a further reduction in sample size. The following combinations were examined: (1) gait and stance (mean annual increase ± SD, 0.51 ± 1.60 points), (2) gait, stance, sitting, and speech (mean annual increase ± SD, 0.97 ± 1.98 points), (3) gait, stance, sitting, speech, fast alternating hand movements, and heel‐shin slide (mean annual increase ± SD, 1.41 ± 2.42 points). Compared with the full set of SARA items, only the third combination was found to require a lower number of patients (ie, 247 vs. 304 per trial arm to detect a 50% reduction in progression). Finally, similar analyses were performed for each of the SCAFI tests, which resulted in considerably larger sample sizes because of higher interindividual variability.

## Discussion

Combining a cross‐sectional and longitudinal approach, this study aimed to comprehensively delineate the temporal dynamics of single SARA items, as well as axial and appendicular subscores, in SCA3 patients, which yielded several important findings. First, axial and appendicular SARA items followed distinct patterns of progression. Finger chase and nose‐finger tests not only had the lowest variability at baseline, but also exhibited the least decline at 1‐year follow‐up. Despite the substantial heterogeneity in disease severity, only a handful of individuals had scores higher than 2 points at both items. Notably, the average nose‐finger test score decreased at follow‐up, which would counterintuitively indicate spontaneous improvement. Alternative explanations could include a possible training effect, fluctuations within a patient, or interrater variability. Second, regarding the choice of the primary endpoint in future therapeutic trials, selection of SARA score without finger chase and the nose‐finger test would require a lower number of patients to detect significant differences than the full set of SARA items or individual SCAFI tests. Third, sex was independently associated with an increase in appendicular subscore and SARA sum score, but not axial subscore, with a faster progression in men than in women. Fourth, annual changes, as expressed by SRMs, were larger for SARA score than each of the SCAFI tests. Except for a weak correlation between change in nose‐finger score and 9HPT performance, there were no clear associations between changes in SARA item scores and corresponding SCAFI tests.

Cross‐sectional estimates of the annual change in SARA score and the observed mean increase after 1 year of follow‐up were nearly identical and in good accordance with the EUROSCA study, which reported a decline of 1.56 points per year.^9^ By contrast, the progression rate of American SCA3 patients in the Clinical Research Consortium for Spinocerebellar Ataxias (CRC‐SCA) study was somewhat slower with a mean yearly increase of 0.65 points.^7^ Although these numbers may suggest a more or less fixed rate of deterioration for every patient throughout the entire disease course, our longitudinal data illustrate a large degree of interindividual variability. While 35.9% of patients had an increase of more than 2 points at 1‐year follow‐up, almost one‐fifth had a lower SARA score compared with the baseline assessment, which could in a therapeutic trial easily be misinterpreted as a treatment effect. Despite the interindividual variability, we found an approximately three times higher average yearly decline in SARA score in patients with a disease duration over 10 years than in those within 10 years from onset. This observation is in line with recently published results in SCA2.^22^

Both cross‐sectional and longitudinal findings allude to the possibility of truly distinct progression rates of axial and appendicular ataxia in SCA3 patients, but may also indicate differences in sensitivity between the various items to capture changes (despite similar 1‐point intervals in the scoring). Our graphs and analyses imply that an increase in SARA score from ~10 to 20 points is predominantly driven by axial and speech items with a considerably smaller contribution of appendicular items. Interestingly, similar plateaus in the curves for finger chase and nose‐finger tests were recently described in individuals with Friedreich ataxia, which argues against an SCA3‐specific effect.^13^ SCA3 and Friedreich ataxia patients thus quickly reach 1 point in both upper limb items, after which progression to higher scores occurs at a much slower pace. This is an important observation in light of therapeutic trials, which may select change in SARA sum score as the primary clinical endpoint, and seems consistent with previous MRI and neurophysiological studies showing degeneration of afferent spinal and pontine pathways before involvement of the cerebellum itself in SCA3.^23^, ^24^, ^25^ It also emphasizes the need for (1) finer, more quantitative tests in the assessment and follow‐up of upper limb ataxia that outperform the human eye (eg, body‐worn sensors), (2) disease‐specific aspects in clinical outcome measures, and (3) comparisons between (changes in) SARA item scores and (ataxia‐specific) patient‐reported outcome measures, such as the recently developed PROM‐Ataxia, to evaluate their clinical meaningfulness.^26^ Here, we also determined associations between 1‐year changes in SARA items and changes in corresponding SCAFI tests as “functional” outcome measures, but acknowledge that the latter are also somewhat artificially constructed metrics.

Sex not only influenced the progression of SARA sum score in our cohort, but also affected the specific pattern of decline. Men exhibited a mean deterioration rate of SARA score that was more than twice as high as that of women and a mean deterioration rate of appendicular subscore that was more than five times as high as that of women. Contributions of axial and appendicular items were roughly equal in men, while progression in women was largely attributable to axial items. We are aware of one prospective study in SCA2 patients that similarly showed a more rapid decline in SARA scores in male individuals.^27^ Previous longitudinal investigations in SCA3 patients that used SARA as primary outcome measure, however, did not find such a sex effect on ataxia progression.^7^, ^9^ Female sex was associated with a faster decline in non‐ataxia signs in the EUROSCA study and a higher risk of becoming dependent on walking aids in a retrospective study that quantified disease severity using four disease stages.^28^, ^29^ As of yet, the biological mechanisms underlying possible differences in symptom evolution between men and women remain unknown and replication of this finding is needed. Besides sex, the annual increase in axial and appendicular subscores was negatively affected by the respective baseline scores, which suggests that there may be less room for further worsening in case of higher baseline values. However, the considerable unexplained variability between patients questions the usefulness of those predictors at the individual level.

Sample size calculations showed that more than 300 SCA3 patients are needed per group to detect a 50% reduction in progression of SARA score in a trial with 1‐year follow‐up. Notably, when only considering mildly affected patients, as defined by a SARA score between 3 and 10, the required number decreases by 43% because of lower interindividual variation. In addition, leaving out finger chase and nose‐finger tests was beneficial because it led to a 19% reduction in sample size compared with the full SARA score.

We used SARA as a (widely accepted) proxy to describe the dynamics of axial and appendicular ataxia. Indeed, the scale has recently been designated as the “recommended” instrument for the assessment of cerebellar symptoms in SCAs, Friedreich ataxia, ataxia telangiectasia, cerebellar stroke, and children with brain tumors.^30^ Although we acknowledge that signs in different domains are depicted with varying levels of granularity, we have analyzed the scale as it was developed and validated more than 15 years ago and as it is currently applied in clinical practice and therapeutic trials. Based on the number of response options, relative contributions of gait, stance, and heel‐shin slide to baseline SARA score were higher than expected, whereas those of the other 5 items were lower than expected, compatible with a distinct temporal evolution of ataxic features in SCA3.

The follow‐up duration of only 1 year could be regarded as a limitation of this study. On the other hand, annual change in SARA score was remarkably similar to the value reported in a long‐term investigation with a maximum observation time of 8 years,^9^ and therapeutic trials will not have a much longer follow‐up. Another limiting factor that might have affected the results, yet reflects common clinical practice, is that SARA ratings at follow‐up visits were sometimes done by a different investigator than at baseline. However, interrater reliability in the SARA validation study was very high, with an intraclass coefficient of 0.98.^11^ Finally, the number of individuals with SARA scores between 25 and 30 and disease durations over 20 years was relatively limited, influencing the robustness of data for this cluster of patients.

In conclusion, this study has provided a more detailed understanding of the natural disease course of SCA3 and particularly revealed discordance between the temporal dynamics of axial and appendicular ataxia as measured with SARA. Our findings will help inform the design of clinical trials and new instruments that evaluate ataxia severity, but also illustrate the difficulty to accurately predict disease progression in SCA3 patients at an individual level using clinical, genetic, and demographic factors.

## Author Roles

(1) Research Project: A. Design, B. Patient recruitment. (2) Statistical Analysis: A. Execution, B. Review and Critique. (3) Manuscript Preparation: A. Writing of the First Draft, B. Review and Critique.R.M.: 2A, 3A: S.T.: 2B, 3B: M.L.: 1A, 3B: P.P.: 1B, 3B: L.P.d.A.: 1A, 3B: J.v.G.: 1B, 3B: D.T.: 1B, 3B: J.I.: 1B, 3B: C.O.: 1B, 3B: K.B.: 1B, 3B: H.J.: 1B, 3B: K.R.: 1B, 3B: M.M.S.: 3B: J.A.R.: 1B, 3B: J.H.S.: 3B: J.J.d.V.: 1B, 3B: M.S.: 1B, 3B: L.S.: 1A, 3B: H.G.M.: 1B, 3B: P.G.: 1A, 3B: J.F.: 1B, 3B: T.K.: 1A, 3B: B.v.d.W.: 1A, 3B

## Full financial disclosures of all authors for the previous 12 months

R.M., S.T., M.L., P.P., J.v.G., D.T., J.I., K.B., H.J., K.R., M.M.S., J.H.S., J.J.d.V., and J.F. report no disclosures.

L.P.d.A.'s research group received funding from the European Regional Development Fund through the Regional Operational Program Center 2020, Competitiveness Factors Operational Program (COMPETE 2020), and National Funds through Foundation for Science and Technology (FCT)—projects UID/NEU/04539/2020, BrainHealth2020 projects (CENTRO01‐0145‐FEDER‐000008), ViraVector (CENTRO‐01‐0145‐FEDER‐022095), SpreadSilencing POCI‐01‐0145‐FEDER029716, as well as private funding from PTC Therapeutics, Wave life Sciences, Blade Therapeutics, and Hoffmann‐La Roche AG.

C.O. receives research support from the National Institutes of Health (NIH), National Ataxia Foundation, the Robert and Nancy Hall Fund for Brain Research, and Alector. He is a consultant for Alector and Acadia.

J.A.R. is a participant of advisory boards for Biogen, Roche, and Novartis and received speaking fees from Biogen.

M.S. has received consultancy honoraria from Orphazyme Pharmaceuticals, Janssen Pharmaceuticals, and Ionis Pharmaceuticals.

L.S. serves on a scientific advisory board of Vico Therapeutics.

P.G. is supported by the NIH Research University College London Hospitals Biomedical Research Centre and CRN North Thames. She has received consulting fees from Novartis, Vico Therapeutics, Reata Pharmaceuticals, and Design Therapeutics.

P.G. and H.G.M. work at University College London Hospitals/University College London, which receives a proportion of funding from the Department of Health's National Institute for Health Research Biomedical Research Centre's funding scheme. P.G. received funding from CureSCA3 in support of H.G.M.'s work.

T.K. receives or has received research support from the Deutsche Forschungsgemeinschaft (DFG), the Bundesministerium für Bildung und Forschung (BMBF), the Bundesministerium für Gesundheit (BMG), the Robert Bosch Foundation, the European Union (EU), and the NIH. He has received consulting fees from Biohaven, uniQure, Vico Therapeutics, Roche, and UBC. He has received a speaker honorarium from Novartis and Bayer.

B.v.d.W. receives research support from ZonMw, Hersenstichting, Gossweiler Foundation, Radboud university medical center, and uniQure, receives royalties from BSL–Springer Nature, and has served on a scientific advisory board of uniQure.

## Supporting information


**Appendix S1** Supplementary data.

## Data Availability

Anonymized data will be shared by the corresponding author on reasonable request from a qualified investigator after approval by the executive board of the ESMI Consortium.
