# Supplementary material for: Differential Temporal Dynamics of Axial and Appendicular Ataxia in SCA3
Source: Mov Disord. 2022 Jul 8;37(9):1850–60. doi: 10.1002/mds.29135 (PMC9540189; doi:10.1002/mds.29135)

**Supplementary data**

**A – Supplementary results**

Changes in SARA item scores versus changes in SCAFI tests in SCA3 patients.

**B – Supplementary tables**

**Supplementary Table 1.** Differences between SCA3 patients who only had a baseline visit and those who returned for follow-up.

**Supplementary Table 2.** Theoretically expected and observed contributions of single SARA items and aggregated subscores to SARA sum score in SCA3 patients at baseline.

**Supplementary Table 3.** Relationships between single and aggregated item scores and SARA sum score at baseline in SCA3 patients.

**C – Supplementary figures**

**Supplementary Figure 1.** Annual change in SARA sum score, axial subscore, and appendicular subscore versus disease duration and the respective baseline (sub)score in SCA3 patients.

**A – Supplementary results**

**Changes in SARA item scores versus changes in SCAFI tests in SCA3 patients**

There was no association between delta walking speed, as measured by the 8MWT, and delta SARA gait (*r* = -0.04, *p* = 0.71) or delta SARA sum score (*r* = 0.07, *p* = 0.51). Similarly, a decline in SARA speech was not paralleled by a worse performance on the PATA repetition task (*r* = 0.09, *p* = 0.28). Concerning the upper limb items, change in time required to complete the 9HPT was weakly but significantly correlated with change in nose-finger score (*ρ* = 0.25, *p* = 0.004) but not with change in fast alternating hand movements score (*ρ* = 0.12, *p* = 0.18). Finally, a somewhat paradoxical relationship was found between worsening of SARA finger chase score and improvement of 9HPT performance and vice versa (*ρ* = -0.16, *p* = 0.071).

**B – Supplementary tables**

|  | **Patients with only a baseline visit (n = 67)** | **Patients with a baseline visit and follow-up (n = 156)** | **P value** |
| --- | --- | --- | --- |
| **Age** | 51.1 ± 11.5 | 51.2 ± 11.1 | 0.95 |
| **Sex (% male)** | 52.2% | 50.6% | 0.83 |
| **Disease duration (y)** | 12.0 ± 6.7 | 11.5 ± 7.0 | 0.65 |
| **SARA score** | 15.0 ± 7.7 | 13.4 ± 7.1 | 0.15 |
| **SARA axial** | 8.0 ± 4.8 | 7.1 ± 4.3 | 0.18 |
| **SARA appendicular** | 5.1 ± 2.5 | 4.5 ± 2.3 | 0.11 |
| **SARA upper limb** | 3.3 ± 1.7 | 2.9 ± 1.7 | 0.22 |

**Supplementary Table 1.** Differences between SCA3 patients who only had a baseline visit and those who returned for follow-up. Results are presented as mean ± standard deviation.

|  | **Expected contribution to SARA sum score** | **Observed contribution to SARA sum score** | **Mean difference (95% CI)** | **P value** |
| --- | --- | --- | --- | --- |
| **Single items** |  |  |  |  |
| Gait | 0.20 | 0.295 | 0.095 (0.084 to 0.106) | < 0.001 |
| Stance | 0.15 | 0.181 | 0.031 (0.021 to 0.041) | < 0.001 |
| Sitting | 0.10 | 0.045 | -0.055 (-0.061 to -0.049) | < 0.001 |
| Speech | 0.15 | 0.121 | -0.029 (-0.039 to -0.019) | < 0.001 |
| Finger chase | 0.10 | 0.083 | -0.017 (-0.025 to -0.009) | < 0.001 |
| Nose-finger | 0.10 | 0.055 | -0.046 (-0.052 to -0.039) | < 0.001 |
| Diadochokinesia | 0.10 | 0.091 | -0.009 (-0.017 to -0.001) | 0.025 |
| Heel-shin slide | 0.10 | 0.13 | 0.030 (0.021 to 0.039) | < 0.001 |
|  |  |  |  |  |
| **Aggregated subscores** |  |  |  |  |
| Axial | 0.45 | 0.521 | 0.071 (0.056 to 0.086) | < 0.001 |
| Upper limb | 0.30 | 0.228 | -0.072 (-0.085 to -0.059) | < 0.001 |
| Appendicular | 0.40 | 0.358 | -0.042 (-0.058 to -0.026) | < 0.001 |
| Total | 1 | 1 | N/A | N/A |

**Supplementary Table 2.** Theoretically expected and observed contributions of single SARA items and aggregated subscores to SARA sum score in SCA3 patients at baseline. Expected contributions are based on the weight of a particular item (e.g., gait 8/40 = 0.20).

|  | **SARA sum score** | | | | | |  |
| --- | --- | --- | --- | --- | --- | --- | --- |
|  | | **3-8 (n = 56)** | **8.5-13.5 (n = 74)** | **14-19 (n = 37)** | **19.5-24.5 (n = 33)** | **25-30 (n = 23)** | |
| **Single items** | |  |  |  |  |  | |
| Gait | | 1.80 ± 0.62 | 2.91 ± 0.91 | 5.22 ± 1.18 | 6.52 ± 0.76 | 7.61 ± 0.50 | |
| Stance | | 1.02 ± 0.73 | 1.89 ± 0.82 | 2.92 ± 1.01 | 4.39 ± 1.03 | 5.65 ± 0.49 | |
| Sitting | | 0.13 ± 0.33 | 0.42 ± 0.52 | 0.92 ± 0.49 | 1.30 ± 0.47 | 2.43 ± 0.99 | |
| Speech | | 0.68 ± 0.79 | 1.28 ± 0.71 | 2.05 ± 0.94 | 2.94 ± 0.86 | 3.70 ± 1.06 | |
| Finger chase | | 0.56 ± 0.46 | 0.95 ± 0.53^*^ | 1.18 ± 0.58^*#^ | 1.50 ± 0.48^#‡^ | 1.78 ± 0.64^‡^ | |
| Nose-finger test | | 0.27 ± 0.37 | 0.66 ± 0.54^*^ | 0.84 ± 0.49^*#^ | 1.15 ± 0.66^#‡^ | 1.39 ± 0.62^‡^ | |
| Diadochokinesia | | 0.55 ± 0.51 | 1.00 ± 0.64^*^ | 1.37 ± 0.77^*^ | 2.09 ± 0.80^‡^ | 2.33 ± 0.68^‡^ | |
| Heel-shin slide | | 0.91 ± 0.58 | 1.49 ± 0.53^*^ | 1.85 ± 0.86^*#^ | 2.18 ± 0.86^#‡^ | 2.89 ± 0.85^‡^ | |
|  | |  |  |  |  |  | |
| **Aggregated subscores** | |  |  |  |  |  | |
| Axial | | 2.95 ± 1.07 | 5.22 ± 1.41 | 9.05 ± 1.79 | 12.21 ± 1.62 | 15.70 ± 1.30 | |
| Upper limb | | 1.38 ± 0.69 | 2.60 ± 1.07 | 3.38 ± 1.16 | 4.74 ± 1.18^*^ | 5.50 ± 0.84^*^ | |
| Appendicular | | 2.30 ± 0.90 | 4.10 ± 1.33 | 5.23 ± 1.63 | 6.92 ± 1.58 | 8.39 ± 1.10 | |

**Supplementary Table 3.** Relationships between single and aggregated item scores and SARA sum score at baseline in SCA3 patients. SARA sum score is grouped in five bins of equal width. Data are expressed as mean scores and standard deviations.

^*#‡^ indicates non-significant differences between consecutive bins (*p* > 0.0125).

**C – Supplementary figures**

**Supplementary Figure 1.** Annual change in SARA sum score, axial subscore, and appendicular subscore versus disease duration and the respective baseline (sub)score in SCA3 patients (A-F), fitted with LOESS regression. Associations are separately shown for men (G-L) and women (M-R). Larger dot sizes indicate overlapping data points.


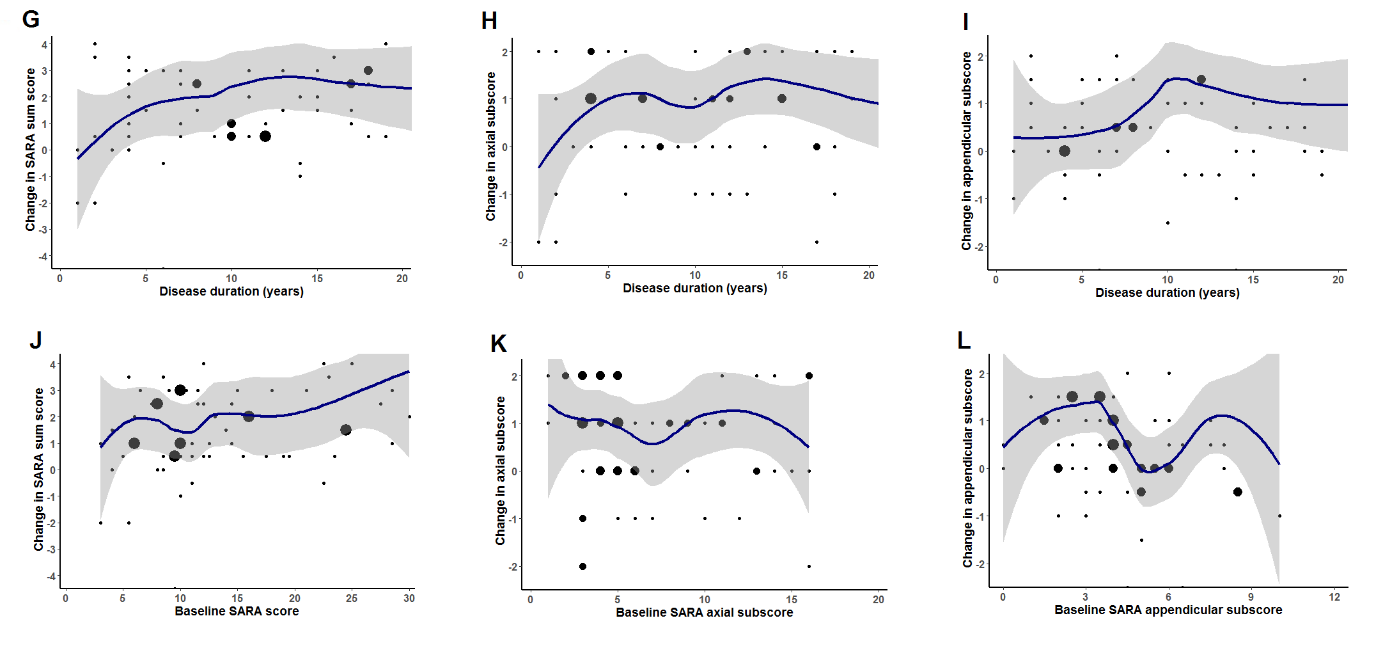

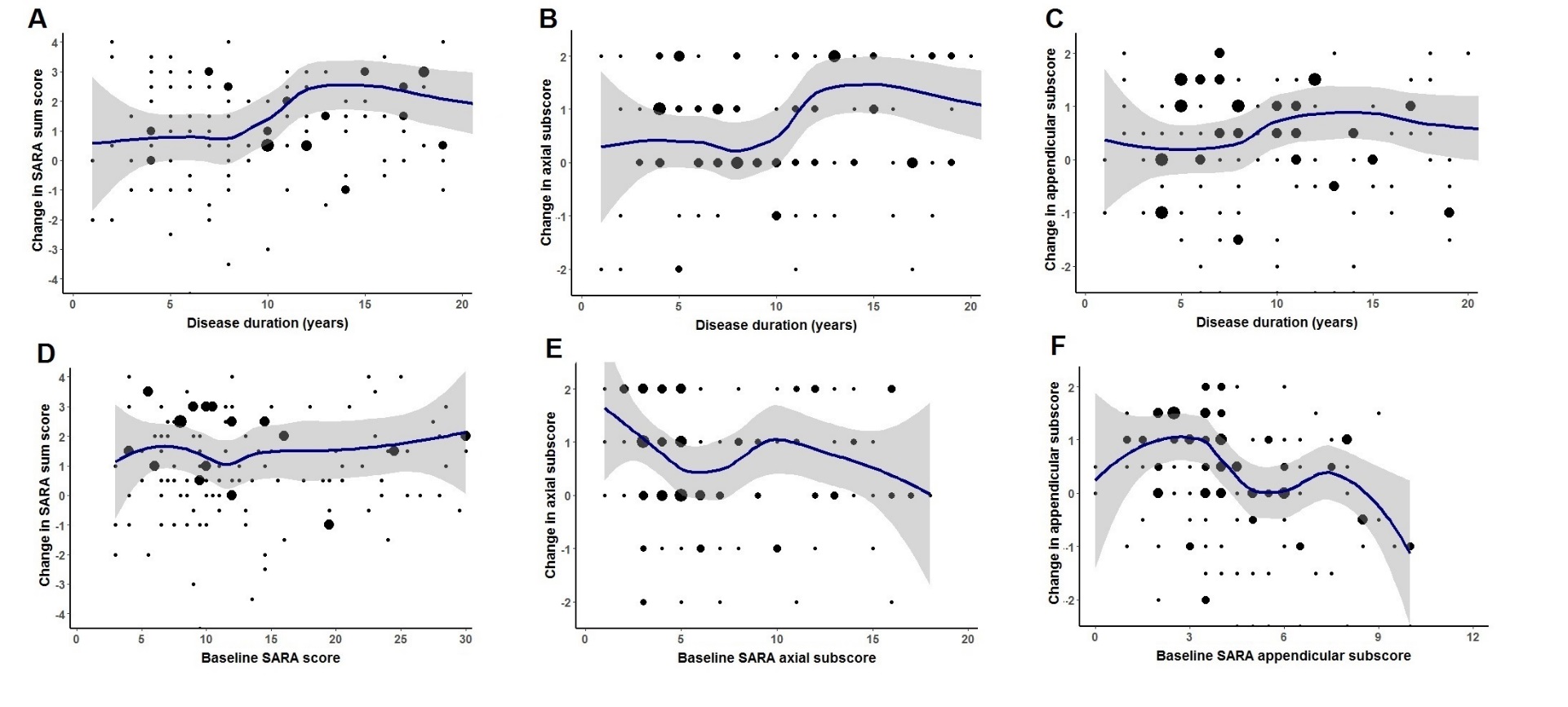


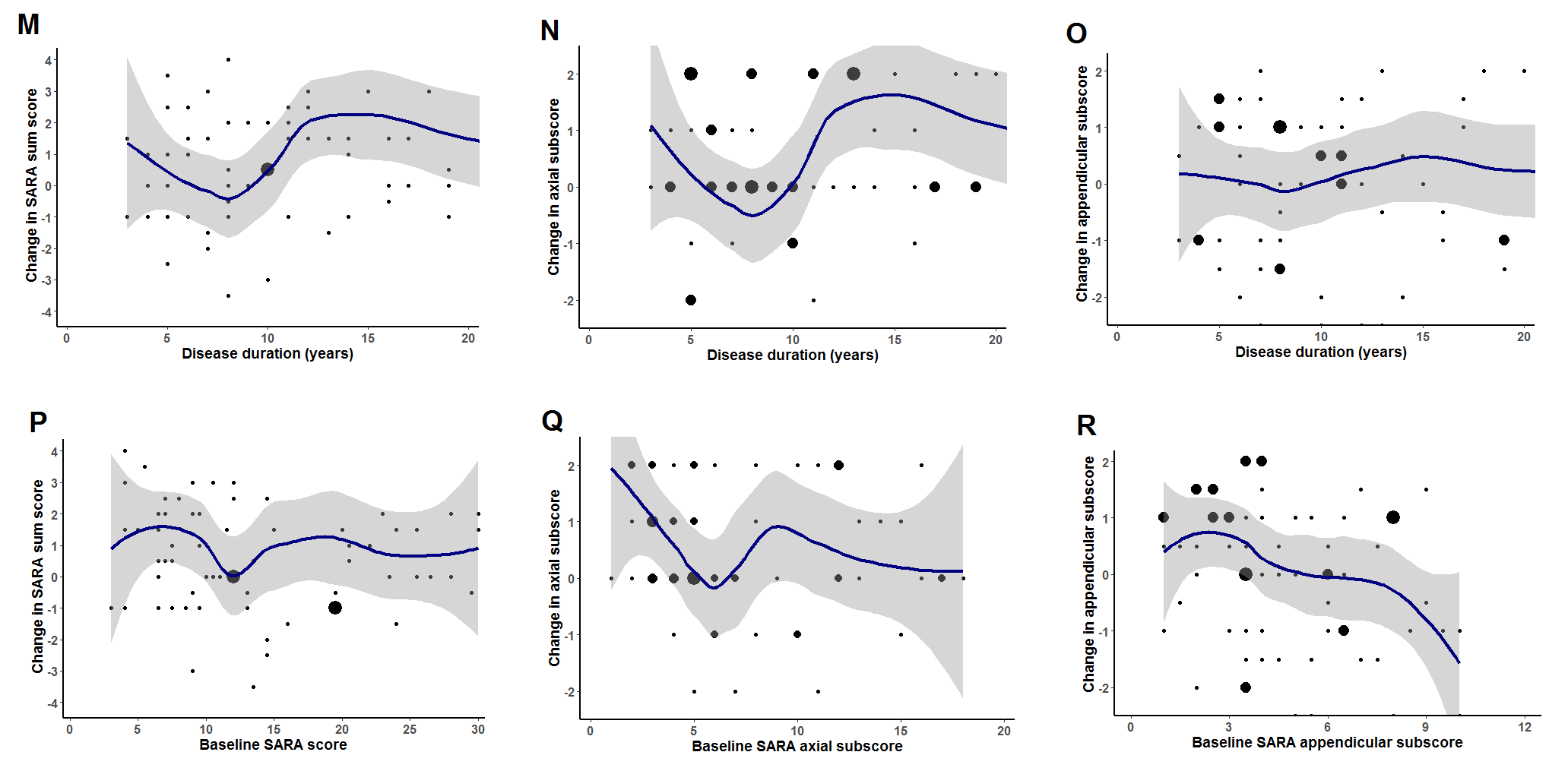

Supplement: Supplementary file 1 — Appendix S1 Supplementary data. [file MDS-37-1850-s001.docx]
